# Musical Competence is Predicted by Music Training, Cognitive Abilities, and Personality

**DOI:** 10.1038/s41598-018-27571-2

**Published:** 2018-06-15

**Authors:** Swathi Swaminathan, E. Glenn Schellenberg

**Affiliations:** 1 Department of Psychology, University of Toronto Mississauga, Mississauga, L5L 1C6 Canada; 2 Faculty of Music, University of Toronto, Toronto, M5S 2C5 Canada

## Abstract

Individuals differ in musical competence, which we defined as the ability to perceive, remember, and discriminate sequences of tones or beats. We asked whether such differences could be explained by variables other than music training, including socioeconomic status (SES), short-term memory, general cognitive ability, and personality. In a sample of undergraduates, musical competence had positive simple associations with duration of music training, SES, short-term memory, general cognitive ability, and openness-to-experience. When these predictors were considered jointly, musical competence had positive partial associations with music training, general cognitive ability, and openness. Nevertheless, moderation analyses revealed that the partial association between musical competence and music training was evident only among participants who scored below the mean on our measure of general cognitive ability. Moreover, general cognitive ability and openness had indirect associations with musical competence by predicting music training, which in turn predicted musical competence. Musical competence appears to be the result of multiple factors, including but not limited to music training.

## Introduction

Musical engagement is widespread, yet individuals vary in musical ability. Some of this variance stems from learning by way of music listening and formal training in music. The remainder stems from natural ability, or musical *aptitude*, which may interact with learning. In the present investigation, we use the term *musical competence* to describe listeners’ ability to perceive, remember, and discriminate musical melodies and rhythms. Unlike *aptitude*, *ability*, or *talent*, the term *competence* is meant to be neutral with respect to the relative roles of nature and nurture.

On most tests of musical competence^1–4^, listeners decide on each trial whether standard and comparison musical sequences are identical. On trials where the sequences differ, one event in the comparison sequence is altered in pitch or time. Although these tests have a long history^5^, few studies have examined how individual differences—other than in music training—predict performance. The goal of the present study was to examine whether variables other than music training can help to predict individual differences in musical competence.

Tests of musical competence are used in pedagogical contexts to identify individuals, particularly children, who are likely to benefit from music training and become skilled musicians. In other words, the tests are often thought to measure “innate” differences in musical ability. In line with this view, empirical studies document a genetic contribution to the perceptual skills relevant to music and the propensity to engage in musical activities. For example, playing music—typically considered to represent an environmental factor—has a substantial genetic component, as does the link between playing and musical ability^6^. In short, the association between music training and musical competence may be—at least in part—an artifact of genetics. Nevertheless, other scholars consider musical competence in general, and expert levels of performance in particular, to be determined almost solely by training and practice^7^. Indeed, music training predicts good performance on tests of musical competence^8–10^, although the direction of causation is unclear. Natural musical ability could determine who takes music lessons, which could, in turn, improve competence. Either way, positive associations with music training provide evidence for a test’s validity^2–4^.

In the present study, we assumed that individual differences in performance on a test of musical competence were a consequence of natural *and* learned musical ability. Musical competence was operationally defined as performance on a standardized test^4^ that measured listeners’ ability to detect a difference between two sequences of tones or beats. In line with the nature and nurture perspectives described above, positive associations between musical ability and music training were expected to reflect both gene-environment correlations (i.e., people seek out environments that match their genetic predisposition) and gene-environment interactions (i.e., effects of experience are moderated by predispositions)^11,12^.

In addition to measuring musical competence and music training, we considered general factors and traits that are likely to be associated positively with musical competence and music training^5^, including general cognitive ability (nonverbal intelligence, working memory, short-term memory), personality (openness-to-experience), and socio-economic status (SES). The predictive power of these additional nonmusical variables was tested with music training held constant. In sum, our goals were to examine: (1) whether musical competence is associated with music training after accounting for nonmusical variables, and (2) whether musical competence is associated with nonmusical variables after accounting for music training. We also tested the possibility that music training might mediate or moderate associations between musical competence and other predictor variables.

Many scholars believe that music training has positive consequences that extend beyond the ability to perform music^13,14^. In line with this view, music training predicts good performance not only on tests of musical skills^15,16^, but also on tests of pitch perception (e.g., frequency discrimination)^16,17^, speech perception (e.g., perceiving speech in noise)^18^, higher-level language abilities (e.g., reading and vocabulary)^19–22^, spatial skills^19^, and general cognitive ability (e.g., processing speed; short-term, working, and long-term memory, intelligence)^19,23–27^. The vast majority of these associations came from studies with correlational or quasi-experimental designs^5^, however, which preclude determination of causal direction. In principle, music training could be causing the associations. Alternatively, individual differences in musical ability, listening skills, and general cognition could be determining who takes music lessons.

In fact, it is well documented that general cognitive ability (e.g., intelligence and working memory) has a strong genetic component^28^. General cognitive ability is also associated positively with music aptitude^4,10,25^, as it is with music training. In some instances, cognitive ability at one point in time predicts music training at a later point, which precludes the possibility that music training caused the observed association^29–31^. Music lessons may go on to strengthen cognitive skills^32^, but this effect appears to be relatively small^10^.

As with general cognitive ability, personality has a strong genetic component^28^. One dimension from the Five-Factor Model of personality^33^, specifically *openness-to-experience* (henceforth, *openness*), is also associated with learning music^23,34,35^ *and* with musical expertise^35,36^. Openness is a marker of aesthetic sensitivity and intellectual engagement^37^. Greater degrees of openness may increase the likelihood of enrollment in music lessons, which then promote the development of listening skills measured by tests of musical competence^35^. Individuals who are high in openness also tend to be more sensitive to music in general^38^, and more likely to use music for cognitive stimulation rather than emotional regulation^39^. Thus, openness could enhance performance on tests of musical competence by increasing interest and motivation.

Finally, we measured socioeconomic status (SES), which is associated positively with participation in extracurricular activities, including music lessons^10,23,26,27^. Higher SES increases the likelihood of being able to afford music lessons, which could then enhance musical competence.

## Results

Preliminary correlational analyses (Table 1) revealed results that were consistent with expectations. More specifically, musical competence tended to be better among participants with more music training. Nevertheless, musical competence also tended to be better among participants from higher-SES families, those who performed well on the measures of short-term memory and general cognitive ability, and those who scored higher on openness. In short, musical competence was associated with demographics, cognitive abilities, and personality. Other simple associations replicated results from previous research^23^: duration of music training tended to be longer as SES increased, as general cognitive ability improved, and as scores on our measure of openness increased. Finally, as general cognitive ability improved, so did short-term memory.

**Table 1:** Simple Associations Among Study Variables.

	1	2	3	4	5
1. Musical competence	—				
2. Music training	0.495*	—			
3. SES	0.283*	0.315*	—		
4. Short-term memory	0.252*	0.108	0.21	—	
5. General cognitive ability	0.410*	0.348*	0.151	0.446*	—
6. Openness to experience	0.340*	0.339*	0.103	0.103	0.067

*Note*. *p < 0.05 (two-tailed).

In the principal analysis, we used multiple regression to model musical competence as a function of music training, SES, short-term memory, general cognitive ability, and openness. Summary statistics are provided in Table 2. Music training, general cognitive ability, and openness each made independent contributions to the model, but SES and short-term memory did not. In contrast to previous research^35^, openness had a direct association with musical competence.

**Table 2:** Multiple Regression Predicting Musical Competence.

Predictor	*β*	*pr*	*sr* ^2^	*p*-value
Music training	0.295*	0.298	0.062	0.007
SES	0.119	0.137	0.013	0.224
Short-term memory	0.064	0.07	0.004	0.535
General cognitive ability	0.246*	0.251	0.043	0.025
Openness to experience	0.205*	0.234	0.037	0.037
Multiple *R*	0.605			<0.001
Multiple *R*^2^	0.367			
Adjusted Multiple *R*^2^	0.326			
*F*(5, 78)	9.028*			

*Note. pr* = partial correlation, *sr*^2^ = squared semi-partial correlation.

The regression model accounted for 36.7% of the variance in musical competence. Music training accounted for 6.2% after the four nonmusical variables were held constant, whereas the four nonmusical variables accounted for 12.2% after music training was held constant. Half of the variance in musical competence that could be explained (18.3%) was accounted for jointly by overlap between music training and the nonmusical variables.

### Mediation analyses

We then tested whether some of this shared variance reflected mediation effects, such that music training mediated associations between musical competence and general cognitive ability (general cognitive ability→music training→musical competence) or openness (openness→music training→musical competence). We used Sobel’s test^40^, and a bootstrap-estimation approach with 50,000 samples^41^ and the PROCESS macro for SPSS^42^. An indirect effect was deemed to be evident if Sobel’s test was significant *and* if the 95% bootstrap confidence interval (CI) for the standardized coefficient of the indirect effect did not contain 0.

In the first test, general cognitive ability was the independent variable, musical competence was the dependent variable, music training was the mediator, and SES and short-term memory were covariates. There was an indirect effect (i.e., through music training; CI: [0.032, 0.229]) from general cognitive ability to musical competence, *p* = 0.020. In the second test, openness was substituted for general cognitive ability. The indirect association through music training was again significant (CI: [0.040, 0.484]), *p* = 0.022. In short, music training partly mediated the association between general cognitive ability and musical competence, and between openness and musical competence. A third test of a mediated effect from SES to musical competence (SES→music training→musical competence; openness, general cognitive ability and short-term memory were held constant) fell short of statistical significance (CI: [−0.004, 0.234]), *p* = 0.064.

It is also possible that the association between music training and musical competence was mediated by general cognitive ability or openness. In other words, music training could enhance general cognitive ability (music training→general cognitive ability→musical competence) or openness (music training→openness→musical competence), which then leads to better performance on the test of musical competence. When we tested these hypotheses, however, neither indirect effect was significant, *p*s > 0.09.

### Moderation analyses

We also asked whether the association between music training and musical competence was moderated by general cognitive ability. For example, music training might be particularly effective at increasing musical competence for participants with below-average (or above-average) levels of cognitive ability. We formed a two-way interaction term between music training and general cognitive ability (after centering the main effects) and tested it in a multiple-regression model that also included the five main-effect predictors. The interaction was significant, *β* = −0.197, *pr* = −0.236, *p* = 0.036, and increased predictive power to 40.2% (adjusted *R*^2^ = 0.355). Follow-up analyses included separate examination of individuals who scored below the mean on general cognitive ability (*n* = 40) and those who scored above the mean (*n* = 44). For participants who scored below the mean, music training had a positive partial association with musical competence, *β* = 0.525, *pr* = 0.544, *p* = 0.001 (SES, short-term memory, and openness held constant), which was relatively strong. For high-scorers, music training had no partial association with musical competence, *p* > 0.3.

We conducted additional analyses to determine whether the association between music training and musical competence was moderated by SES, *p* > 0.9, or openness, *p* > 0.5, but no additional moderation effects were found.

## Discussion

Performance on our test of musical competence was associated positively with duration of music training, SES, short-term memory, general cognitive ability, and openness. A linear combination of the five predictors accounted for over a third of the variance in musical competence, with music training, general cognitive ability, and openness explaining variance independently of each other and of SES and short-term memory. Although music training had the strongest simple and partial associations with musical competence, the nonmusical variables (considered jointly) explained a larger portion of the variance. The association between music training and musical competence was also partly due to the fact that high-functioning and open individuals had an increased likelihood of taking music lessons for longer durations of time.

Although the association between music training and musical competence was strong among participants who scored relatively low on our measure of general cognitive abilities, it was negligible among participants who scored high. Indeed, participants with above-average ability tended to perform well on the test of musical competence whether or not they had music lessons. These findings suggest that (1) musical engagement is most effective in training musical abilities for those with low performance on tests of general cognitive ability, or (2) long-duration music training among such individuals is particularly dependent on pre-existing listening skills. Either way, the results could have practical relevance for music educators.

Others have reported that general cognitive ability is associated with basic music perception^4,25^. Our findings confirmed that the association is not simply an artifact of SES, openness, or music training. Openness was also associated with musical competence when music training, general cognitive ability, and SES were held constant. One previous study^35^ reported a similar mediated association between openness and musical competence (through music training), but no direct association. Thus, our finding of a direct association is novel, and consistent with the proposal that openness is a marker of musical motivation and interest that predicts performance on tests of musical competence, irrespective of music training. These results are also consistent with previous reports^23,43^, which indicate that the role of personality should be considered whenever researchers ask whether individual difference in music training or musical competence are associated with other variables.

Our behavioral results have striking parallels with those from twin studies, which document that performance on musical-competence tests is moderately heritable, such that a music-specific genetic factor (i.e., independent of genetic factors explaining intelligence) accounts for almost a third of the variance in scores on tests of musical competence, whereas approximately 40% is explained by the same genetic factors that explain general cognitive ability^44^. Common genetic factors also underlie intelligence and the propensity to take music lessons^45^. In short, genetic predispositions influence musical competence *and* musical engagement. The present findings provide converging behavioral evidence that musical competence is *not* simply the outcome of learning to play music.

As noted, music training had the strongest simple and partial associations with musical competence, but the nonmusical variables (considered jointly) explained a larger portion of the variance. If music training has far transfer effects, as some have suggested^14^, one would expect the causal evidence for near transfer to be particularly strong. Nevertheless, in the relatively few longitudinal studies with random assignment to music-training or control groups, the results are inconclusive. The most convincing evidence comes from interventions that emphasize *rhythm* training and/or listening skills. Such training enhances phonological awareness among young children^46^, which predicts reading ability. Moreover, successful rhythm-based interventions have been reported for children with dyslexia or poor reading ability^47–49^. One intensive (i.e., 5 days/week for 4 weeks) computer-based program for preschoolers led to larger improvements in vocabulary compared to a control group that had training in visual art^21^. The music training focused almost exclusively on listening, however, and the children did not learn to play an instrument or sing.

When musical interventions are more like typical instrumental or singing lessons, however, the results tend to be weaker. For example, when 8-year-olds were assigned to 2 years of music or painting training, after 1 year the music group had stronger neural responses to syllables that differed in duration or voice-onset time, even though no behavioral differences were evident^50^. After 2 years, the music group had better behavioral performance and stronger neural responses on a task that required them to segment strings of syllables^51^. During initial learning of the strings, however, syllables were matched one-to-one with different pitches, which likely provided a more beneficial cue to children with music training. In a similar but shorter-term study (6 months)^20^, children who studied music were better than children who studied painting on tasks that required them to identify prosodic anomalies in speech, or to read irregularly spelled words. Nevertheless, the anomaly in prosody was created by shifting the pitch of a syllable (thereby favoring the music group), and music-training advantage was evident on only one of three reading measures.

In another study, 6- to 9-year-olds from low-income families were assigned randomly to an established music program, or to a control group that received no training of any sort^52^. After 1 year, the music group did not show the typical *decline* in reading performance that was evident in the control group. After 2 years, the music group exhibited enhanced performance at perceiving speech in noise, but overall attrition by this time was over 50% and the samples were very small (*n*s < 20)^53^. Moreover, adults with many years of music training can show no advantage over untrained adults on similar tests of perceiving speech in noise^54,55^. Such inconsistent findings may arise from unmeasured individual differences that influence who takes music lessons.

In any event, although rhythm and listening training may be beneficial for individuals with reading problems, there is limited evidence for causal effects of more typical music lessons on listening skills, speech, or language ability. Moreover, most of the positive evidence comes from correlational studies (for reviews see^14,56^), perhaps due in part to the fact that there are far fewer experimental studies with longitudinal designs. In any event, the question of causation remains open. This issue is especially problematic because musical competence predicts speech perception better than music training does^9^, and the association between music training and general cognitive ability is primarily genetic in origin^45^. After many years of research on transfer effects, we know that *near* transfer (to a closely related domain or skill) is more common than *far* transfer (to an unrelated or distantly related domain or skill). Thus, if music lessons have a limited effect on musical competence (near transfer), as our findings suggest, their effect on nonmusical abilities (far transfer) would almost certainly be much smaller.

One limitation of the present study is that musical competence was defined solely as performance on a 20-min test. Future research could benefit from operationalizing musical competence in different or multiple ways, perhaps with the *Goldsmiths Musical Sophistication Index*^57^, which places greater emphasis on musical interest and involvement, or the *Profile of Musical Perception Skills*^2^, which has additional subtests. For example, the association between music training and musical competence could be stronger when motivation plays a stronger role, or evident for some aspects of musical ability but not for others. Another limitation is that our study design was correlational. Nevertheless, the results inform future longitudinal studies with random assignment, which could benefit from measuring musical, cognitive, and personality abilities at the outset. For example, tests of moderation effects in longitudinal designs could reveal that music training may be particularly effective when higher (or lower) levels of musical competence, cognitive ability, and/or openness are evident before the intervention.

In sum, our findings indicate that the association between music training and performance on a test of musical competence arises from complex interactions between nature and nurture. Future investigations of experience-dependent plasticity would benefit from employing designs that can capture such complexity. Here, we demonstrated that realistic but nuanced interpretations can be gleaned from correlational data when nonmusical individual differences are considered jointly with music training. Although “practice makes perfect” is considered to be the basis for expertise in music and other domains, our results reveal this to be an oversimplification. In general, musical competence was also better among individuals with good cognitive skills and open-minded personalities. Although music training was a good predictor of musical competence for participants with low performance on tests of cognitive abilities, it had negligible predictive power for participants with high performance. Successful replication of this finding in experimental contexts could have important implications for music educators.

## Method

The study protocol was reviewed and approved by the Research Ethics Board at the University of Toronto, the methods were carried out in accordance with the relevant guidelines and regulations, and informed consent was obtained in writing from all participants.

### Participants

Participants were 84 undergraduates (60 female) whose average age was 19.1 years (*SD* = 2.1). Sample size was determined from previous research^9,10^, which indicates that the true correlation between music training and performance on our test of musical competence is approximately 0.35 in the population from which our sample was drawn. For a 95% chance of detecting an association of this magnitude, we needed a sample of 79 participants.

On average, participants reported 2.7 years of private music lessons taken outside of school (*SD* = 5.7) and 3.2 years of lessons taken in school (*SD* = 4.4). For statistical analyses, we summed duration of private and school training and applied a square-root transformation to reduce positive skew. Response patterns were unchanged when alternate measures of music training were adopted (see Supplementary Table S1).

### Measures

#### Socioeconomic status

Participants provided information about their parents’ education and family income (as in previous studies^9,10,23^). Mothers’ and fathers’ education were measured separately on scales ranging from 1 (did not complete high school) to 8 (graduate degree) and averaged. Annual family income was measured on a scale ranging from 1 (<$25,000) to 9 (>$200,000). We used principal components analysis to extract a single latent variable (hereafter *SES*), in order to reduce collinearity and measurement error. It accounted for 65% of the variance in the original measures. Six missing values were replaced with the mean.

#### General cognitive abilities

Working memory was measured with the Digit Span subtest from the Wechsler Adult Intelligence Scale^58^. Nonverbal intelligence was measured with the abbreviated 12-item version of Raven’s Advanced Progressive Matrices^59^. Because our test of musical competence required same-different judgments, it is a test of short-term memory for musical stimuli, such that performance is correlated with other measures of short-term memory^4,9,60^. Accordingly, raw scores on the forward portion of Digit Span (*short-term memory*) were considered separately and included in the analyses to ensure that musical competence was more than just auditory short-term memory. We extracted a principal component (*general cognitive ability*) from the Advanced Progressive Matrices and the backward portion of Digit Span, which accounted for 61% of the variance in the original scores.

#### Personality

The Big Five Inventory^61,62^ was used to measure personality. Although we administered the entire test to maintain its psychometric properties, we included only openness in the analyses because we did not expect other personality traits to be relevant^23^. Indeed, the four other personality dimensions were *not* associated with other measured variables (see Supplementary Table S2).

#### Musical competence

The 20-min Musical Ear Test^4^ is a measure of musical competence that comprises 104 same-different trials. It was computer-administered with stimuli presented over headphones. On each trial, participants heard two short auditory sequences and judged whether they were identical. On the Melody subtest (administered first), sequences comprised three to eight piano tones. On the Rhythm subtest, participants heard two sequences of 4–11 beats of a woodblock. On “different” trials, one of the tones or beats was altered in pitch (Melody) or time (Rhythm). The principal component was extracted from the number of correct responses on the two subtests (*musical competence*). It explained 72.1% of the variance in the subtests.

### Procedure

Participants were tested individually in a quiet room. They completed the Advanced Progressive Matrices, followed by the Musical Ear Test, Digit Span, a background questionnaire, and the Big Five Inventory. The entire testing session took approximately 75 min. Fifty-three participants completed the Advanced Progressive Matrices and Musical Ear Test as part of another study and were invited back to complete the Digit Span, background questionnaire, and Big Five Inventory.

### Data availability

The data are available in Supplementary Materials.

## Electronic supplementary material

Supplementary Material
