# Supplementary material for: Musical Competence is Predicted by Music Training, Cognitive Abilities, and Personality
Source: Sci Rep. 2018 Jun 15;8:9223. doi: 10.1038/s41598-018-27571-2 (PMC6003980; doi:10.1038/s41598-018-27571-2)
Supplement: Supplementary file 1 — Supplementary Material [file 41598_2018_27571_MOESM1_ESM.pdf]

Musical Competence is Predicted by Music Training, Cognitive Abilities, and Personality

Swathi Swaminathan[1], and E. Glenn Schellenberg[1, 2 *]

[1]Department of Psychology, University of Toronto Mississauga, Mississauga, L5L 1C6, Canada

[2]Faculty of Music, University of Toronto, Toronto, M5S 2C5, Canada

*g.schellenberg@utoronto.ca

Supplementary material

Table S1

Associations of Study Variables with Music Training Coded in Multiple Ways

		Square-root duration	Duration	Training vs. no training	Three-level coding†
1.	Musical competence	.495**	.341**	.474**	.444**
2.	SES	.315**	.294**	.188*	.321**
3.	Short-term memory	.108	.069	.110	.141
4.	General cognitive ability	.348**	.300**	.318**	.344**
5.	Openness	.339**	.330**	.192*	.238**

Note. * Indicates significance (one-tailed), ** indicates significance (two-tailed).

† Participants were coded as 0 for those reporting no training, 1 for those reporting school-based *or* private lessons, and 2 for those reporting school-based *and* private lessons.

Table S2

Associations of Personality Traits with Study Variables

	Openness	Conscientiousness	Extraversion	Agreeableness	Neuroticism
1. Musical competence	.340**	-.064	.044	-.098	-.018
2. Music training	.339**	-.091	-.112	-.089	.088
3. SES	.103	-.115	-.108	-.141	-.130
4. Short-term memory	.103	-.003	.037	-.131	-.104
5. General cognitive ability	.067	-.178	-.061	-.159	.001

Note. * Indicates significance (one-tailed), ** indicates significance (two-tailed).
